# A Triple-Isotope Approach to Predict the Breeding Origins of European Bats

**DOI:** 10.1371/journal.pone.0030388

**Published:** 2012-01-23

**Authors:** Ana G. Popa-Lisseanu, Karin Sörgel, Anja Luckner, Leonard I. Wassenaar, Carlos Ibáñez, Stephanie Kramer-Schadt, Mateusz Ciechanowski, Tamás Görföl, Ivo Niermann, Grégory Beuneux, Robert W. Mysłajek, Javier Juste, Jocelyn Fonderflick, Detlev H. Kelm, Christian C. Voigt

**Affiliations:** 1 Leibniz Institute for Zoo and Wildlife Research, Berlin, Germany; 2 Environment Canada, Saskatoon, Saskatchewan, Canada; 3 Estación Biológica de Doñana (Consejo Superior de Investigaciones Científicas), Sevilla, Spain; 4 Department of Vertebrate Ecology and Zoology, University of Gdańsk, Gdańsk, Poland; 5 Faculty of Veterinary Science, Institute for Biology, Szent István University, Budapest, Hungary; 6 Nature Conservation Foundation of Tolna County, Szekszárd, Hungary; 7 Leibniz Universität Hannover, Institut für Umweltplanung, Hannover, Germany; 8 Groupe Chiroptères Corse, Corte, Corsica, France; 9 Association for Nature “Wolf”, Lipowa, Poland; 10 Department of Biology and Ecology, Montpellier SupAgro, Montpellier, France; 11 Centre d'Ecologie Fonctionnelle et Evolutive, Montpellier, France; 12 Department of Biology, Cognitive Neurobiology, Humboldt University Berlin, Berlin, Germany; University of Bern, Switzerland

## Abstract

Despite a commitment by the European Union to protect its migratory bat populations, conservation efforts are hindered by a poor understanding of bat migratory strategies and connectivity between breeding and wintering grounds. Traditional methods like mark-recapture are ineffective to study broad-scale bat migratory patterns. Stable hydrogen isotopes (δD) have been proven useful in establishing spatial migratory connectivity of animal populations. Before applying this tool, the method was calibrated using bat samples of known origin. Here we established the potential of δD as a robust geographical tracer of breeding origins of European bats by measuring δD in hair of five sedentary bat species from 45 locations throughout Europe. The δD of bat hair strongly correlated with well-established spatial isotopic patterns in mean annual precipitation in Europe, and therefore was highly correlated with latitude. We calculated a linear mixed-effects model, with species as random effect, linking δD of bat hair to precipitation δD of the areas of hair growth. This model can be used to predict breeding origins of European migrating bats. We used δ^13^C and δ^15^N to discriminate among potential origins of bats, and found that these isotopes can be used as variables to further refine origin predictions. A triple-isotope approach could thereby pinpoint populations or subpopulations that have distinct origins. Our results further corroborated stable isotope analysis as a powerful method to delineate animal migrations in Europe.

## Introduction

Bats are the only mammals to have conquered the aerospace by the evolution of powered flight, an ability which has allowed them to colonize almost all habitats worldwide. Some species have adapted to urbanized environments and live in close proximity to humans, and yet the feeding and roosting ecology of many bats remain enigmatic and their movements difficult to assess due to their small size and their cryptic and nocturnal habits. Like birds, bats can move to new habitats more readily than most terrestrial mammals. However, unlike birds, evidence of long-distance movement in bats was not documented until the mid-20th century, when the use of mark-recapture (banding) in bats started to yield first results [1].

More than one million bats have been banded in Europe over the past 80 years [2]. Recaptured bats have provided valuable information on migratory connectivity (i.e. the movement of individuals between summer and winter populations, including immediate stopover sites [3]) and on general patterns of movement directions [2], but for most species only anecdotal data with strong geographical bias has been obtained. Overall, the probability of documenting long-distance movements by recapturing banded bats, like for many small species, has proven to be very low [2]. The potential harmful effects of banding on bat populations (e.g. forearm lesions, injuries, reduced fitness or hunting success) have prompted some countries to abandon bat banding [2], [4]–[5]. Unfortunately few other technological alternatives exist to document individual bat movements directly [6]. The smallest satellite transmitters available at present (approx. 2g) are still too heavy for most temperate-zone bat species. In addition, these miniature transmitters do not include an option for remote downloading or for tracking via built-in VHF transmitters, and thus require “blindly” recapturing the animal to retrieve the data, a highly unlikely event in the case of a migratory bat.

As a result, little remains known about which bat species perform long-distance migrations in Europe, their migration routes, and possible population-specific differences in migratory strategies. This is a fundamental gap of knowledge in bat ecology that limits the efficiency of conservation efforts, since identification of both wintering and breeding regions is critical for the protection and long-term persistence of bat populations [7]. All European bat species are protected by the Habitats Directive 92/43/CEE (Annexes II and IV), and are included under the Convention on the Conservation of Migratory Species of Wild Animals (Bonn, 1979) through the EUROBATS Agreement (London, 1991; implemented since 1994), regardless of their migratory behavior. The rationale behind this legislation is that all bat species are subject to the same threats, mostly habitat degradation, contamination through pesticides and disturbance of roosting sites, and the fact that roosts are often shared by migratory and non-migratory species (CMS, 1991; http://www.cms.int/species/eurobats/bat_text.htm). Moreover, all temperate-zone bats move between summer and winter roosts. Depending on the extent of migratory behavior, European bat species have been classified as long-distance migrants, regional migrants, and sedentary, but some species are also described as occasional migrants or vagrants [2]. Information on movements between summer and winter roosts of banded bats reveal that there is no clear-cut separation between these groups regarding movement distances [2], so that ascribing a species to a particular migratory category is somewhat arbitrary based on current knowledge. Within a single species, individuals with different migratory strategies may even coexist in the same areas [8].

The discovery that the stable isotopic composition of animal tissues reflects the isotopic ratios of local water and food sources motivated scientists to use the large-scale isotope patterns present in the hydrosphere and terrestrial landscapes (isoscapes) to quantify movement magnitudes and link breeding and wintering areas of migratory animals, such as birds, butterflies, fish and elephants and even humans [9]–[11]. Since isotopic signatures in animal tissues are endogenous markers, no recapture of the same individual is needed. For animals, stable hydrogen isotopes (δD) in seasonally-grown inert tissues (e.g. hair, feathers) are considered especially useful to track the locations of tissue growth, since strong, systematic and predictable isotopic gradients exist in meteoric waters across continents, mostly with a latitudinal component, driven by hydrological and meteorological processes [12]–[14]. The δD composition of local water is incorporated into local food webs and fixed in newly-formed animal tissues. Keratinous tissues such as feathers and hair retain this isotopic “fingerprint” even after the animal moves to a new location, since they do not change chemically after synthesis. It has been shown for both North America and Europe that δD values in bird feathers and insect tissues grown at known locations strongly correlate with δD of local precipitation water [15]–[19]. The relationship between δD in keratinous tissues and rainfall has been used to estimate origins of migratory butterflies and birds, for which tissue formation time and molt chronology were previously known [20]–[23].

Few studies have applied δD analysis to the study of bat migration. In a pioneering work, Cryan et al. [24] used the hydrogen isotope approach to study the migration of the hoary bat *Lasiurus cinereus* sampled throughout North America. They calculated the relationship between δD in precipitation water (δD_p_) and δD in bat hair (δD_h_) using data collected in different seasons. Because no previous studies using hair of bats of known origin had been conducted, they assumed that net isotopic discrimination between hair and local water was −25‰, as previously reported in birds. They estimated the period of hair molting as the time when the difference between δD_p_ and δD_h_ was closest to this value. The results supported the general observation that bats molt once per annum during summer at their breeding areas [24], [25]. They computed the relationship between δD_p_ and δD_h_ using only data from the defined molting period, and used this regression to estimate migratory distances. Britzke et al. [26] investigated the relationship between δD_p_ and δD_h_ for four North American bat species, one long-distance migrant and three regional migrants. As δD_h_ in migrants do not necessarily reflect δD_p_ of the sampling location, they collected hair during the breeding period when the bats are presumed to be sedentary and growing new hair. They found a weak relationship between δD_h_ vs. δD_p_, and δD_h_ vs. latitude for most species and sex/age categories. Moreover, the regressions were inconsistent among species and categories. Inter-individual and inter-specific differences in molting times and fidelity to the breeding areas were proposed as some of the reasons that possibly led to these inconsistent results [26].

To apply hydrogen isotopes to the study of bat migration in Europe, it must first be firmly established that δD_p_ or some other geographical covariate (e.g. latitude, longitude) of the location of hair formation are good predictors of δD_h_ of European bats. We chose to test this relationship using hair of sedentary bat species with a relatively broad distribution across Europe. In sedentary species that spend the summer and winter at nearby roosts (<50 km apart), the isotopic composition of hair should reflect that of local water and diet, so samples were collected at any time of the year. We developed a linear mixed effects model of δD_h_ vs. δD_p_ for bats of known origin with species as random effect, and further tested the relationship of δD_h_ with other geographical covariates. It was anticipated that robust spatially calibrated regression models could later be used to estimate breeding origins of migratory bats. Since multiple isotope tracer approaches have the potential to better assign individuals to origin [27], [28], we investigated whether δ^13^C and δ^15^N could help further discriminate among sampling sites that overlapped in their δD values. Based on our results, we discussed the potential and limitations of stable isotopes as a means for inferring breeding origins of European migratory bats.

## Methods

### Sampling of selected species

Sampling throughout Europe was conducted by an international network of bat scientists who collected hair samples for this study during their own bat research activities. Sampling took place from 2005–2009. The bats were captured either at roosts or in foraging areas, or occasionally, hair was taken from bats found wounded or dead, e.g. dead bats found under wind turbines. All persons responsible for hair collection were qualified and experienced bat researchers who had bat capture and sampling permits issued by the competent environmental authority of their study regions.

We selected hair samples from five bat species reported to have sedentary habits: *Barbastella barbastellus*, which rarely covers distances of more than 20 km between summer and winter roosts [2]; *Eptesicus serotinus*, for which summer and winter roosts are usually less than 40–50 km apart [2]; its sibling, allopatric and ecologically similar species *Eptesicus isabellinus* occurring in Southern Iberia [29], with no documented long-distance movements from banded individuals in Southern Spain, similar home ranges as *E. serotinus* and forming extremely philopatric maternity colonies [30], [31]; and *Plecotus auritus* and *P. austriacus*, both with seasonal movements in the range of a few kilometers [2]. A small amount of hair (less than 5 mg) was cut with small scissors from the interscapular region of the bats' back. Hair was stored in a small plastic tube and kept at ambient temperature until preparation for isotopic analysis.

### Stable isotope analysis

All stable hydrogen, carbon and nitrogen isotope analyses of bat hair were conducted at the stable isotope lab of the Leibniz Institute for Zoo and Wildlife Research (IZW) in Berlin.

#### Hair standards for δD analysis

For δD analysis, we used the comparative equilibration method described by Wassenaar and Hobson [32], in which samples were analyzed together with previously calibrated keratin hydrogen isotope reference materials. This method overcomes the problem of uncontrolled hydrogen isotopic exchange between keratin and isotopically variable ambient moisture in the laboratory.

We prepared five in-house hair standards, collected from locations that spanned the latitudinal range of bat sampling sites: sheep from Sweden (SWE-SHE), sheep from Germany (GER-SHE), sheep from Spain (ESP-SHE), horse from Spain (ESP-HOR) and goat from Tanzania (AFR-GOA). Each hair type was obtained from only one animal and came thus from a single location.

Hair laboratory standards were washed to remove dirt, dried and cleaned of surface oils by soaking for 24 hours in a 2∶1 chloroform∶methanol solution, decanted and dried again in a fume hood to remove the solvent. 50 g of each standard type were powdered using a Freezer Mill 6800 (C3 Prozess- und Analysentechnik GmbH, München, Germany). The resulting powder was sieved to a particle diameter size of <63 µm (VS 1000, Retsch, Haan, Germany) and blended to ensure best possible homogeneity of the standards. These five lab standards were analyzed at the Stable Isotope Laboratory of Environment Canada, Saskatoon, 5 times each using the offline steam equilibration method using the keratin references for normalization described in detail in Wassenaar and Hobson [32], [33]. This procedure measured the isotopic value of the non-exchangeable hydrogen (δD_n_). δD_n_ (mean ± SD) of the hair standards were: SWE-SHE: −167.9±1‰; GER-SHE: −136.8±1.5‰; ESP-SHE: −108.3±1‰; ESP-HOR: −98.7±0.5‰; AFR-GOA: −66.3±0.9‰. All hair standards had similar exchangeable hydrogen properties (8–10%).

#### Preparation of standards and samples for comparative equilibration in δD analysis

Bat hair samples were solvent cleaned in a 2∶1 chloroform∶methanol solution and dried in the same way as the standards, but were not powdered, as this would be impractical due to long processing times; this procedure is similar to that long used for feathers [34]. Bat hairs and homogenized standards were weighed using a microbalance (Sartorius ME5, Göttingen, Germany) to a target weight of 1100±10 µg of hair, which was transferred into 4×6 mm silver-foil capsules (IVA Analysetechnik e.K. Meerbusch, Germany). These were folded into tiny cubes and stored in 96-well microtiter plates loosely covered with the lid, to allow ambient exchange with air moisture. The trays were left for at least one week on the shelf of the lab to allow samples and standards to equilibrate with air moisture, or placed in a compartment drier at 50°C for at least 24 h to speed up equilibration and remove extra moisture. The samples and hair standards were then immediately loaded into the carrousel of a Zero Blank autosampler (Costech Analytical Technologies Inc., Italy) and flushed with helium for 1 h. We used three, sometimes four different hair lab standards in each autosampler batch (autorun).

#### Stable hydrogen isotope analysis

The standards and bat samples were pyrolized in a high-temperature elemental analyzer (HTO Elementaranalysator HEKAtech GmbH, Wegberg, Germany) operating at 1450°C and containing a silicon carbide (SiC) tube filled halfway with glassy carbon chips. Helium was used as carrier gas with a flow rate of 100 ml/min. The gas chromatograph, operating at 70°C, separated H_2_, N_2_, and CO. The resolved H_2_ sample pulse was introduced into the isotope ratio mass spectrometer (Delta V Advantage IRMS, ThermoFischer Scientific, Bremen, Germany). Isotopic ratios, corrected for instrumental drift, were expressed in typical delta notation as units per mill (‰) and normalized on the VSMOW-SLAP standard scale. Analytical precision based on the repeated analyses of unequilibrated powdered hair standards was ±1.02‰. All bat hair samples were analyzed in duplicate. When the difference between the two measurements of one individual was greater than 10‰, two further analyses were performed. If no outlier was detected, the mean value of the four measurements was used for the statistical analyses.

#### δ^13^C and δ^15^N analysis

For δ^13^C and δ^15^N analysis, samples were weighed to a target weight of 0.35±0.05 mg and placed into 4×6 mm tin capsules. Samples were combusted using a Flash EA 1112 Series elemental analyzer (Thermo Italy, Rhodano, Italy) and the carbon and nitrogen stable isotope ratios of the combusted organic material were measured using a Delta V Advantage isotope ratio mass spectrometer (Thermo Finnigan, Bremen, Germany) operating in continuous flow mode. Carbon and nitrogen isotope ratios were reported in the delta notation in units per mill (‰) relative to V-PDB or AIR-N_2_ scales, respectively. Our laboratory standards (tyrosine and leucine) were calibrated with the international standards NBS 19, NBS 22, USGS 24 and L-SVEC for carbon and IAEA-N-1, IAEA-N-2 and IAEA-NO-3 for nitrogen.

### Statistical treatments

We used Arc GIS 9.3.1 to extract δD of mean annual precipitation values (δD_p_) associated with the UTM coordinates of the hair sample locations (Table S1) from 10′×10′ resolution grid maps developed by Bowen [35]. We modeled the relationship between δD_h_ and possible explanatory variables (see below) using R.2.10.1 [36]. For all analyses we set the significance level for the *P*-value at 0.05. We first calculated a correlation matrix (Pearson's product moment correlation *r*) between all variables (δD_p_, δD_h_, δ^13^C, δ^15^N, latitude, longitude and elevation) to get a first impression of their interrelation. We then developed two different models to test for spatial dependencies, (a) for δD_h_ from bats of known origin vs. δD_p_ and (b) another model disentangling the relationship of δD_h_ with geographical covariates latitude, longitude and elevation. (a) To assess the appropriate model structure, we used a likelihood ratio test (L) for comparing a fixed effects model fitted by generalized least squares (GLS) with two mixed effects models, one containing a random intercept, and one with random intercept and slope (package nlme) following Zuur et al. [37] (Table S2). All models were estimated with restricted maximum likelihood (REML). We calculated the *P*-values based on L using the correction provided by Verbeke and Molenberghs [38] for comparing models without random effects versus models with random intercept, and for comparing models with random intercept versus models with random intercept and slope. We used the optimal random structure to fit the final model for regressing δD_h_ vs. δD_p_. (b) We then used the single spatial variables longitude, latitude and elevation including their three-way interaction and calculated a mixed effects model with species as random intercept. Before entering the independent variables, we used generalized additive models with three knots (GAM; package mgcv) to visually check the linearity assumption of the variables, including species as a fixed factor. Non-linear variables were included as quadratic term. For both models, we finally used the Kolmogorov-Smirnov normality test with Lilliefors correction to test for homogeneity in residuals (package nortest).

We further evaluated the utility of a triple isotope approach (δD, δ^13^C and δ^15^N) to discriminate potential origins using only *Eptesicus* sp., i.e. the species group with the largest sample size. We removed sampling locations for which number of individuals was <4, and tested with a multivariate analysis of variance (MANOVA) in SPSS Statistics v. 19 for differences in δD, δ^13^C and δ^15^N between all remaining locations. We performed discriminant function analysis (DFA) to test whether the location of origin for a particular hair sample could be predicted based on the triple isotopic assays (performed with SPSS Statistics v. 19). DFA was used to assign samples (in this case, individual hair samples) to groups that were defined in advance (in this case, all sampling locations included in the analysis), and generated linear combinations of the variables (δD, δ^13^C and δ^15^N) that were then used to separate the groups based on minimizing distances (in our analysis, the Mahalonobis distance was used) among the samples within groups [39]. The results of the classification gave the probability of correctly assigning samples to a group. As sample size was insufficient to partition the samples in two groups, one for developing the model and the other one for validating it, we performed a jackknife classification, also called “leave-one-out cross-validation” (all samples except one were used to create the model, which was then used to classify the excluded sample).

## Results

**Figure 1:**
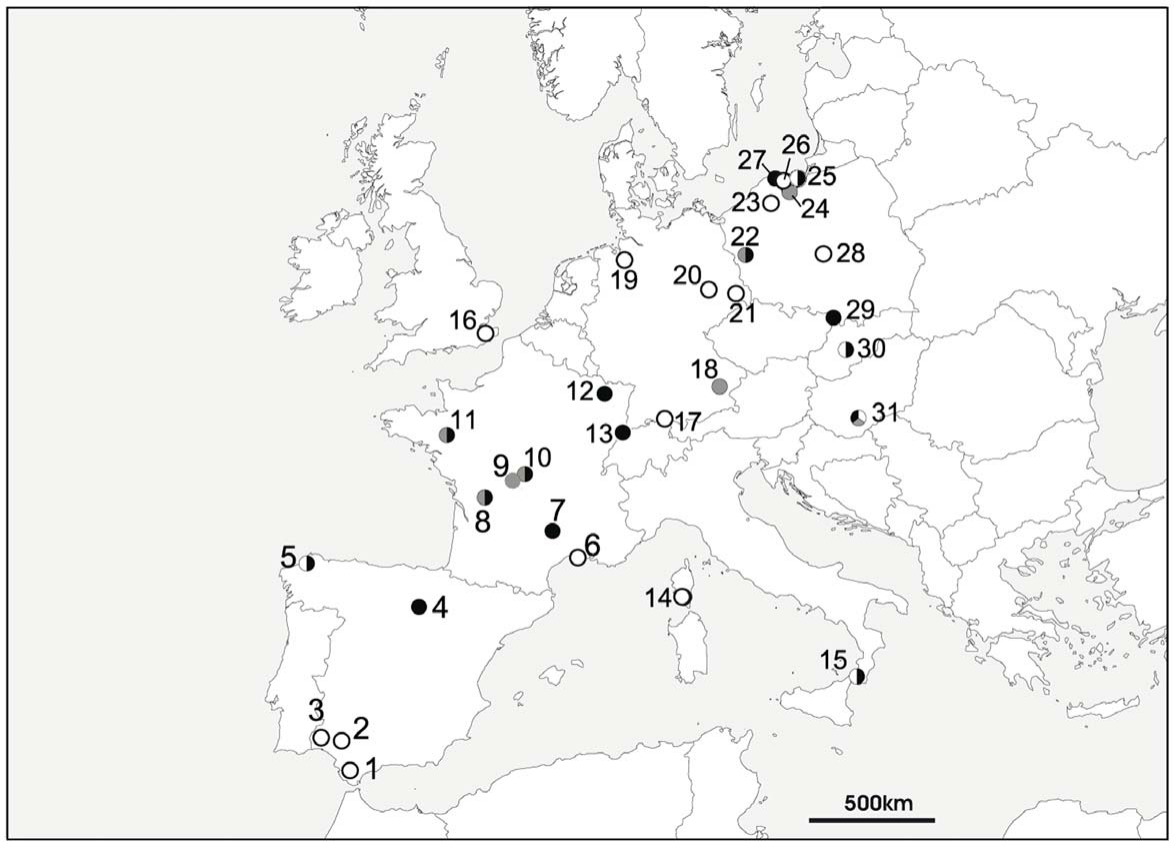
Site locations of bat hair samples. Circles are filled in according to the species or genus at that site: *Eptesicus serotinus*/*E. isabellinus*, open circles; *Barbastella barbastellus*, grey circles; *Plecotus auritus*/*P. austriacus*, black circles. Proximate sampling sites were grouped as one circle for clarity. Numbers refer to sites in Table S1.

We used hair samples from 178 bats captured at 45 sites throughout Europe (Table S1, Figure 1; grouped into 31 sites for simplicity of representation). δD_h_ values ranged from −40 to −105‰ (Table S1). The difference between two δD_h_ measurements of the same individual ranged from 0.03 to 19‰, but was ≤4‰ for 84% of all sampled individuals of the five species. Variance within a population (inter-individual) ranged between 2.7 and 12.0‰ (SD; Table S1).

**Figure 2:**
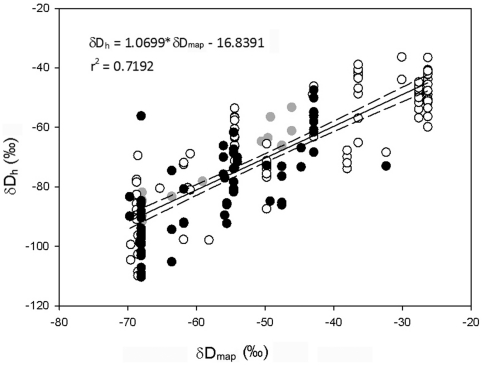
Correlation between δD of bat hair (δD_h_) and δD of mean annual (δD_p_) precipitation. Regression (line), 95% confidence intervals (dashed) and regression equation are given for the whole data set (n = 178). Empty circles: *E. serotinus/E. isabellinus*; black circles: *Plecotus auritus/P. austriacus*; grey circles: *Barbastella barbastellus*.

On a first examination of the data, we found strong (Pearson's *r*>0.7) significant correlations between δD_h_ of sedentary bats and δD_p_ (Pearson's *r* = 0.85, Figure 1, Figure 2), latitude and longitude (correlation matrix, Figure S1). δ^13^C also correlated with δD_p,_ latitude and longitude (Figure S1). δ^15^N showed a significant but weak correlation with elevation (Pearson's *r* = 0.20). Among the three isotopic markers, δD_h_ and δ^13^C were significantly correlated; δ^13^C and δ^15^N were weakly but significantly correlated (Pearson's *r* = 0.18; Figure S1).

(a) The likelihood ratio test showed that the model containing species as random intercept performed best, indicating that adding a random effect species to the model yielded significant improvement (model a1, *P* = 0.03; Table S2). In the resulting linear mixed-effects model, δD_p_ had a significantly positive effect on δD_h_ (*P*<0.0001; Table 1). (b) Visual inspection of the single geographic variables in the GAM revealed that only elevation was non-linear, and consequently it was included as a quadratic term (Figure S2). The linear mixed-effects model for predicting δD_h_ from latitude, longitude, and elevation, with species as random intercept showed that latitude, longitude, and their interaction had a significant effect on δD_h_. Elevation and its interaction with the other variables had no significant effect on δD_h_ (Table 2). The residuals of both models were normally distributed (see Tables 1 and 2).

**Table 1:** Results of the linear mixed-effects model fit by reduced maximum likelihood (REML; model a1 in Table 2) for predicting δD_h_ from δD_p_, with species as random intercept.

Model parameter	Estimate	SE	df	t-value	*P*
Intercept	−17.21	3.11	172	−5.54	<0.0001
δD_p_	1.07	0.05	172	18.90	<0.0001

Number of observations: 178. Number of groups (random effect species): 5. AIC = 1331.97, BIC = 1334.65, logLik −666.98. The random intercept was normally distributed, with mean 0 and standard deviation (SD) 2.54, and so was its residual term, with mean 0 and SD 9.85. Model residuals were normally distributed (Lilliefors D = 0.0266, *P* = 0.7844).

**Table 2:** Results of the linear mixed-effects model fit by REML (model b) for predicting δD_h_ from latitude, longitude and elevation, with species as random intercept.

Model parameter	Estimate	SE	df	t-value	*P*
Intercept	−4.96	11.31	166	−0.44	0.66
Longitude	2.02	0.89	166	2.28	0.024
Latitude	−1.26	0.25	166	−5.00	<0.0001
Elevation^2^	0.0	0.0	166	1.14	0.26
Longitude:Latitude	−0.058	0.02	166	−3.13	0.002
Longitude:Elevation^2^	0.0	0.0	166	−1.121	0.26
Latitude:Elevation^2^	0.0	0.0	166	−1.41	0.25
Longitude:Latitude: Elevation^2^	0.0	0.0	166	1.09	0.28

Number of observations: 178. Number of groups (random effect species): 5. AIC = 1444.34, BIC = 1475.69, logLik −712.17. The random intercept was normally distributed (mean 0, SD 5.62), and so was its residual term (mean 0, SD 9.38). Model residuals were normally distributed (Lilliefors D = 0.0403, *P* = 0.1732).

2 : variable included as quadratic term.

**Figure 3:**
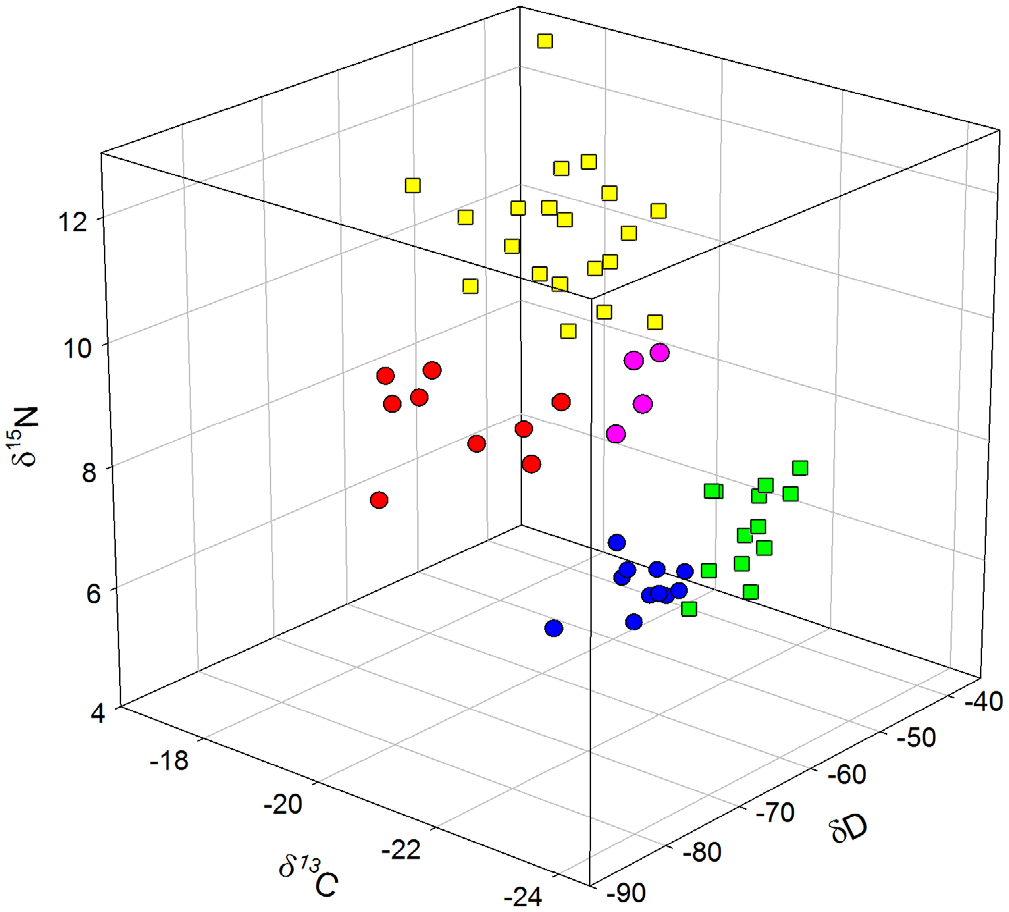
Multi-isotope triplet for *Eptesicus serotinus*/*E. isabellinus*. Circles represent values of *E. serotinus* samples, squares of *E. isabellinus*. Colors represent different sampling sites of hair. Yellow: 2; green: 3; pink: 6; blue: 14; red: 31 (numbers refer to sites in Table S1 and Figure 1).

To test the utility of a triple isotope approach (δD, δ^13^C and δ^15^N) for predicting sample origin of *Eptesicus* sp., we selected five (from 21) capture locations (numbers 2, 3, 6,14 and 31 in Table S1 and Figure 1; n individuals ≥4). Data points from individuals originating from the same location formed distinctive clusters in a 3-D scatter plot (Figure 3), indicating that samples from the same location had a similar triple isotopic profile. Overall, locations were significantly different in their triple isotopic patterns (MANOVA Wilks' Lambda *F_12, 133_* = 59.9, *P*<0.001).

Results of the DFA are shown in Table S3. By means of their triple isotope compositions, 93.0% of the jackknife cross-validated *Eptesicus* sp. individuals were assigned correctly to their sampling location. When using only δD, DFA had an accuracy of 47.4% in assigning samples to site. A combination of two isotopes was sufficient to raise assignment efficiency to almost 90% (89.5% for δD-δ^15^N and 86% for δD-δ^13^C and δ^13^C-δ^15^N).

## Discussion

The lack of suitable tracking methods has so far hampered our understanding of migratory connectivity of European bats, information which is essential for conservation planning. We tested the utility of stable isotopes as geographic tracers of breeding origins of bats in Europe. Our test consisted of validating (ground trothing) the accuracy and precision of hair isotopic signatures for predicting the geospatial origin of bats, by using samples of resident, known-origin bats captured in their natural habitat. We then built a regression model that may serve to predict region of hair growth for migrating bats from their hair isotopic ratios.

We can find various examples in the literature of different approaches to ground truthing isotopic endogenous markers for studying animal migration. One approach is to breed the animals or keep them in captivity during the period of tissue growth in several locations scattered over the geographic range of interest, providing the animals only with local water and food. Hobson et al. used this approach to study monarch butterfly migration in North America [17]. While the advantage of the method is that it allows obtaining a specific regression model for the species of interest, it is impractical for most species, and in particular for insectivorous bats due to the difficulty in keeping and feeding these animals. Another approach is to sample animals in the wild throughout the desired range at the time and place of tissue formation, before the animals move to a new location. For example, Kelly et al. computed the relationship between latitude and δD of feathers of migratory Wilson's warblers captured at the breeding areas, where feathers are replaced [40]. This approach has the limitation that good knowledge of molting phenology is required. Although bats are known to molt in summer, the exact phenology and its variation between individuals and sexes is not known, nor is the degree of between-year fidelity to the summer areas. These problems are exemplified in a study that tested the relationship between δD of hair and δD of local precipitation or latitude for several North American, long-distance or regional migratory bat species [26]: although hair was collected during the breeding season when the bats were presumed to be sedentary and hair to be grown locally, the relationship between δD in hair and δD of precipitation (or latitude) was very weak.

Our approach was to use samples from bat species known to have sedentary habits throughout its European distribution range. We thus followed Hobson et al. [19], who computed the relationship between δD of feathers and δD of precipitation using samples from resident birds in Europe, and later used this relationship to predict origins of migratory woodpigeons [23]. This approach is nevertheless not devoid of pitfalls, since, as mentioned previously, there is no clear-cut separation between migratory and non-migratory bats. Temperate bats have separate summer and winter roosts, and a large number of species are considered regional migrants, at least potentially. We decided to restrict our analysis to five species for which the scientific body of evidence points towards sedentary habits [2], [30], [31], [41].

Resident European bats, like birds [19], strongly reflected δD of local precipitation in their keratinous tissues, in this case hair. Similarly to European resident birds, δD_h_ of resident bats correlated strongly with δD_p_ (*r* = 0.85, Figure S1). This correlation was higher in our study than in resident European birds (*r* = 0.81, [19]) and North American migratory bats sampled during summer (*r* = 0.77, [24]). Given that the δD_p_ data of the maps that we used are partly derived from geographic position (by a method known as “detrended interpolation”, [35]), it was not surprising that δD_h_ also correlated with latitude (*r* = −0.77) and longitude (*r* = −0.74, Figure S1). Latitude and longitude also had an influence on δ^13^C values of bat hair (*r* = −0.72 and *r* = −0.41 respectively, Figure S1), while elevation correlated significantly, but weakly, with δ^15^N (*r* = −0.40). These results are consistent with known but weak global latitudinal patterns in plant δ^13^C values [42]) and with evidence of elevational effects on plant and soil δ^15^N values [43]. Elevation did not correlate with δD_h_ (Figure S1), and it did not improve the linear mixed-effects model linking δD_h_ with latitude and longitude (Table 2). This was surprising, since the depletion of rain water δD with elevations above 300–500 meters above sea level (asl) is a well-known phenomenon that has been used to infer animal movements along elevational gradients [44]. Altitude of our sampling sites ranged from 0 to 1400 m asl (Table S1). We hypothesize that the high mobility of bats and the heterogeneity of their feeding and drinking sites could have masked elevational effects on δD_h_.

We investigated the relationship between δD_h_ of bats and δD_p_ as a proxy of geographic location, with the aim of obtaining a generalized isotopic model that may be used to estimate the location of hair growth, and thus for predicting breeding origins of migrating bats. We used a linear mixed model linking δD_h_ and δD_p_, with species as random intercept, and consequently incorporating the variance associated with species differences. Incorporating species as random slope did not improve the model; this suggested that although different species may show a different offset (net isotopic discrimination factor) between dietary water and body tissue, their δD_h_ changes with δD_p_ in the same manner. In contrast to our results, Britzke et al. found significant interspecific variation in the regression equations of δD_h_ vs. δD_p_, with differences in both slopes and intercepts, of four North American bat species [26]. But contrary to our study the species tested were not resident but one long-distance and three regional migrants, and the relationship between δD_p_ vs. δD_h_ was weak. The model obtained here accounted for interspecific variation in discrimination factors and was therefore not species-specific. Yet, our analysis included only five bat species, and therefore caution should be exercised when extrapolating the model to other species.

Although δ^13^C correlated with latitude and longitude, we did not incorporate δ^13^C in the model because no reliable maps of δ^13^C plant in Europe exist that could be used for geographic assignment. The reason for this is the large variance in δ^13^C plant values, so that local differences generally obscure the rather weak global patterns [34], [42]. Additionally, δ^13^C of bat hair in our study correlated strongly with δD_h_ and therefore these two variables were not fully independent. The large dependency of δ^13^C and δ^15^N on source of diet and trophic level may further limit their predictive value for migration studies when high interspecific and interpopulation differences in diet preferences cannot be ruled out. A trophic level effect on δD of vertebrate tissues has also been suggested [45], particularly for bats [46], [47], but differences have only been found between very distinct trophic levels, such as animalivores vs. herbivores [45], [47] and sanguinivores vs. frugivores [46]. Since all European bats are insectivorous, we do not believe that differences in dietary preferences would speak against the use of multiple-species models for predicting breeding origins of European bats based on δD. δ^13^C and δ^15^N were unsuitable to create spatially predictive geographical models for keratin growth location of wildlife in Europe, since there are no clear geographical patterns of these two isotopes in European habitats. Nevertheless, several studies have shown that isotopic markers that do not reflect clear, predictable geographic patterns can indeed help interpretations on migratory connectivity and behavior [34]. For example, the probability of correct assignment of feathers of known origin of red-winged blackbirds to sampling site improved from 64% to 80% when using δ^13^C values in addition to δD values [18]. Other studies have used a combination of isotopes (mostly δD and δ^13^C, sometimes also δ^15^N or δ^34^S) to test whether different breeding populations of migratory birds stem from the same or different wintering areas [48]–[50], or to determine natal catchment areas of migrating birds sampled en route [51]. We tested the utility of a triple-isotope approach (δD, δ^13^C and δ^15^N) to discriminate origins of hair growth. For this analysis, we did not pool species with different dietary niches because δ^13^C and δ^15^N values are known to be influenced by diet and ecology. However, to achieve an appropriate sample size, isotopic data of *Eptesicus serotinus* and *E. isabellinus* were analyzed together, based on the assumption that these species (until recently considered the same species, [29]) are ecological equivalents. The sampled populations of *Eptesicus* sp. were distinctive using a combination of three isotopes, with almost no overlap among them. The probability of estimating correctly the origin of an unknown sample stemming from one of these locations would be 93%. Admittedly, our analysis included only five locations, so it is possible that with a higher number of locations, an increased overlap would have hampered the predictive value of our approach. Still, the five populations considered included some distant locations, like sites in Spain (sites 2 and 3 in Figure 3 and Table S1) and Hungary (site 31), and other proximate locations, like Seville (site 2) and Huelva (site 3) in Southern Spain (Figure 3 and Table S1). Even these two locations, barely 75 km apart, did not overlap in their triple isotopic delineation.

In bat migration studies, measuring δ^13^C and δ^15^N values (and possibly additional isotopes like ^34^S or ^18^O) may be useful to evaluate whether two or more migrating bat populations that are not significantly distinctive based on δD alone have different breeding origins. This could be the case for males and females exhibiting different migratory strategies, resident and sedentary populations of the same species coexisting in an area, or bats stemming from different breeding populations gathering together at swarming sites or wintering colonies. In addition, it may eventually be possible to use combined triple isotopic values to compare the migratory populations of interest to reference isotopic data from breeding populations to find the most likely population of origin, i.e. the one with the most similar isotopic profile.

Based on our results, we determined that stable isotopes are a promising and powerful tool to estimate the origins of migratory bats in Europe. A predictive model linking δD_h_ of sedentary bats of known origin with δD_p_ was obtained which may be used for translating δD_h_ of migratory bats into δD_p_ of the place of origin. Incorporating sources of error (i.e. intra-population variance) into a GIS-based probability model, potential regions of origin which have δD_p_ values matching those predicted can be identified. The use of δ^13^C and δ^15^N and possibly other isotopic assays in addition to δD may further help to identify origins through direct comparison between migrating populations of interest and their potential breeding counterparts. Stable isotope analysis therefore represents a cost-effective endogenous marker that does not require recapture of marked individuals. Being informative on a population level and relatively non-invasive, it is possibly the best technological method currently available to study bat migration in Europe.

## Supporting Information

Figure S1 **Correlation matrix (Pearson's r) for the variables δD_p_, δD_h_, δ^13^C, δ^15^N, latitude, longitude and elevation.** The variable names are shown in the middle diagonal; *r* values are in the upper right half (* corresponds to a significance level of 0.05, ** 0.01, and *** 0.001); scatter plots are presented in the lower left half.
(TIF)

Figure S2 **Results of the GAM used to test the linearity assumption of the variables latitude, longitude and elevation.** The y-axis shows the partials (k = 3 for smoothing function) of the respective variable. The variable elevation does not show linear behavior and was therefore modeled as quadratic term.
(TIFF)

Table S1 **Sampling locations of bat hair.** Name of location, site number (refers to Figure 1), number of individuals of each species captured at each site, mean stable-hydrogen isotopic ratios (‰ relative to VSMOW-SLAP), ± standard deviations when N>3, per species and site, and site elevation.
(DOC)

Table S2 **Results of the likelihood ratio test (L) for finding the optimal random structure of the linear mixed-effects model (LME) for predicting δD_h_ from δD_p_.** All models were estimated by REML.
(DOC)

Table S3 **Results of the discriminant function analysis to test the utility of a triple isotope approach for discriminating sampling locations of** ***Eptesicus*** **sp.** A. Standardized coefficients given to δD, δ^13^C and δ^15^N to maximize the difference between locations. B. Structure matrix, pooled within-groups correlations between discriminating variables and standardized canonical discriminant functions. Variables ordered by absolute size of correlation within function. *. Largest absolute correlation between each variable and any discriminant function. C. Functions at group centroids; unstandardized canonical discriminant functions evaluated at group means. D. Results of the classification in leave-one-out cross-validation, showing how many cases from each location were predicted to stem from each other location, and the total percentage of correctly classified samples per location. For all locations, 93.0% of cross-validated grouped cases were correctly classified.
(DOC)
