# Supplementary figures and images for: A Triple-Isotope Approach to Predict the Breeding Origins of European Bats
Source: PLoS One. 2012 Jan 23;7(1):e30388. doi: 10.1371/journal.pone.0030388 (PMC3264582; doi:10.1371/journal.pone.0030388)

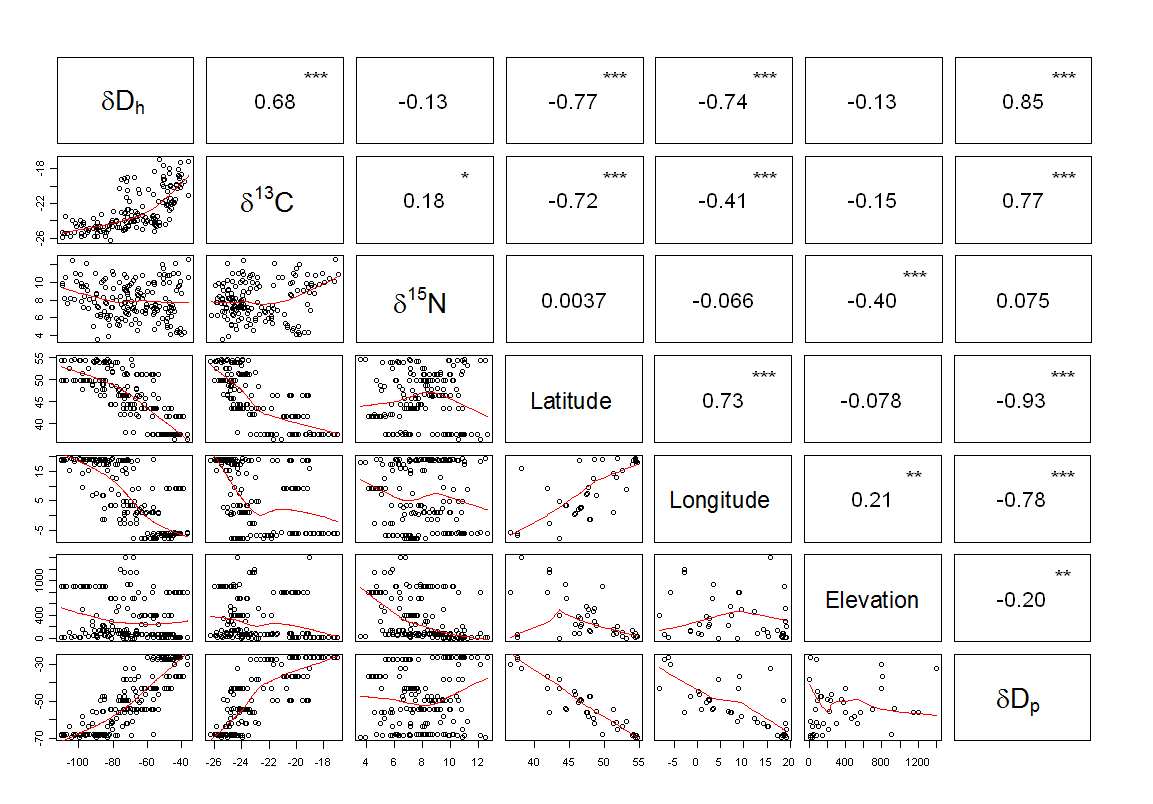

Supplement: Figure S1 — Correlation matrix (Pearson's r) for the variables δDp, δDh, δ13C, δ15N, latitude, longitude and elevation. The variable names are shown in the middle diagonal; r values are in the upper right half (* corresponds to a significance level of 0.05, ** 0.01, and *** 0.001); scatter plots are presented in the lower left half. (TIF) [file pone.0030388.s001.tif]

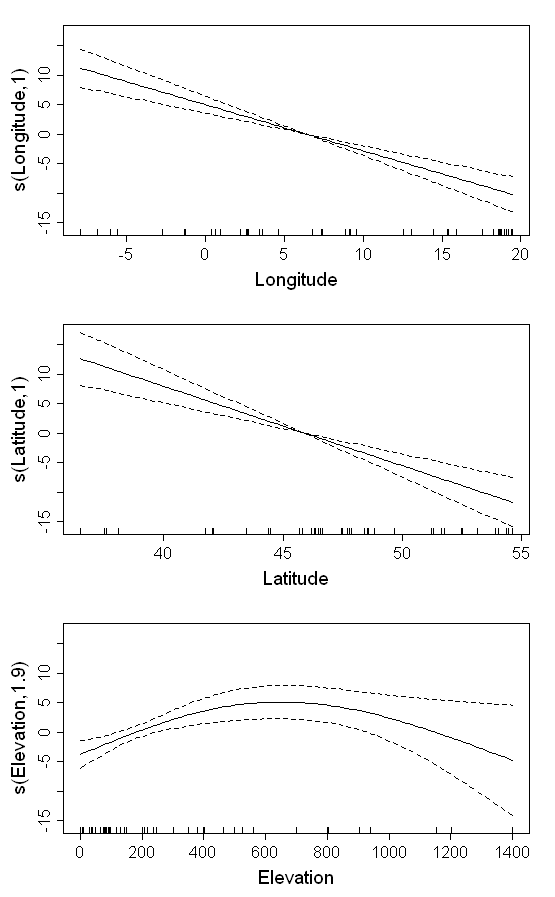

Supplement: Figure S2 — Results of the GAM used to test the linearity assumption of the variables latitude, longitude and elevation. The y-axis shows the partials (k = 3 for smoothing function) of the respective variable. The variable elevation does not show linear behavior and was therefore modeled as quadratic term. (TIFF) [file pone.0030388.s002.tif]
