# Supplementary material for: A Triple-Isotope Approach to Predict the Breeding Origins of European Bats
Source: PLoS One. 2012 Jan 23;7(1):e30388. doi: 10.1371/journal.pone.0030388 (PMC3264582; doi:10.1371/journal.pone.0030388)
Supplement: Table S1 — Sampling locations of bat hair. Name of location, site number (refers to Figure 1), number of individuals of each species captured at each site, mean stable-hydrogen isotopic ratios (‰ relative to VSMOW-SLAP), ± standard deviations when N>3, per species and site, and site elevation. (DOC) [file pone.0030388.s003.doc]

**Table S1.** Sampling locations of bat hair.

| **Site** | **Name** | **N indiv/sp** | **δD_hair_ ± SD (‰)** | **Elevation (m)** |  |
| --- | --- | --- | --- | --- | --- |
| 1 | Cortes de la Frontera, Málaga, Spain | 2 *E. isabellinus* | -40.1 | 800 |  |
| 2 | Alcalá del Río, Sevilla, Spain | 20 *E. isabellinus* | -47.7 ± 5.9 | 10 |  |
| 3 | Calañas, Huelva, Spain | 13 *E. isabellinus* | -49.0 ± 3.4 | 70 |  |
| 4 | Villoslada, La Rioja, Spain | 4 *P. auritus* | -71.3 ± 4.1 | 1150 |  |
| 5 | As Pontes de García Rodríguez, A Coruña, Spain | 2 *E. serotinus*  11 *P. auritus* | -54.7  -56.8 ± 5.6 | 400 |  |
| 6 | Salin de Badon, Camargue, France | 4 *E. serotinus* | -70.9 ± 2.7 | 0 |  |
| 7 | Balsièges, Lozère, France | 7 *P. auritus* | -74.7 ± 8.7 | 700 |  |
|  | Les Bondons, Lozère, France | 1 *P. auritus*  1 *P. austriacus* | -70.1  -71.6 | 940 |  |
| 8 | Rancogne, Charente, France | 2 *P. auritus* | -70.1 | 90 |  |
|  | Lésignac-Durand, Charente, France | 2 *B. barbastellus* | -57.2 | 250 |  |
| 9 | Gouzon, Limousin, France | 1 *B. barbastellus* | -64.7 | 380 |  |
| 10 | Saint-Bonnet-Tronçais, Allier, France | 1 *B. barbastellus* |  | 235 |  |
|  | Nassigny, Allier, France | 1 *P. austriacus* | -84.8 | 200 |  |
|  | Le Brethon, Allier, France | 1 *B. barbastellus* | -56.5 | 350 |  |
| 11 | Forêt d'Araize, Bretagne, France | 3 *P. auritus*  1 *P. austriacus*  1 *B. barbastellus* | -78.5 ± 6.8  -85.3  -66.1 | 130 |  |
|  | Forêt de Corbière, Bretagne, France | 1 *B. barbastellus* | -66.4 | 120 |  |
| 12 | Zommange, France | 3 *P. auritus* | -87.9 ± 3.8 | 220 |  |
| 13 | Kiffis, Alsace, France | 1 *P. auritus* | -77.0 | 560 |  |
|  | Biederthal, Alsace, France | 1 *P. auritus* | -89.5 | 400 |  |
| 14 | Quenza, Corsica, France | 1 *E. serotinus* | -49.0 | 800 |  |
|  | Zonza, Corsica, France | 11 *E. serotinus* | -46.7 ± 7.9 | 800 |  |
| 15 | Diga Sul Menta, Calabria, Italy | 1 *E. serotinus*  1 *P. auritus* | -68.4  -73.0 | 1400 |  |
| 16 | Crundale, Kent, UK | 11 *E. serotinus* | -62.6 ± 6.4 | 70 |  |
| 17 | Bodenseekreis, Germany | 1 *E. serotinus* | -97.9 | 500 |  |
| 18 | Ofen, Bayern, Germany | 1 *B. barbastellus* | -78.1 | 460 |  |
| 19 | Lilienthal, Germany | 2 *E. serotinus* | -75.0 | 5 |  |
| 20 | Elbe-Elster, Germany | *1 E. serotinus* | -80.3 | 80 |  |
| 21 | Bautzen, Germany | 2 *E. serotinus* | -85.1 | 150 |  |
| 22 | Bledzew, Lubuskie Lakeland, Poland | 2 *P. austriacus* | -84.4 | 40 |  |
|  | Nowa Wies, Lubuskie Lakeland, Poland | 1 *P. auritus*  1 *B. barbastellus* | -105.2  -83.2 | 100 |  |
| 23 | Płęsno, Pomerania, Poland | 7 *E. serotinus* | -83.8 ± 4.8 | 140 |  |
| 24 | Czarlin, Pomerania, Poland | 5 *B. barbastellus* | -86.5± 4.0 | 50 |  |
| 25 | Przebrno, Vistula Spit, Poland | 1 *P. auritus* | -98.7 |  |  |
|  | Krynica Morska, Vistula Spit, Poland | 2 *E. serotinus* | -102.0 | 10 |  |
| 26 | Gdańsk-Suchanino, Pomerania, Poland | 1 *E. serotinus* | -96.3 | 80 |  |
|  | Gdańsk, Pomerania, Poland | 3 *E. serotinus* | -102.5 ± 11.6 | 10 |  |
|  | Sopot, Pomerania, Poland | 2 *E. serotinus* | -86.1 | 30 |  |
| 27 | Wejherowo, Pomerania, Poland | 2 *P. auritus* | -86.6 | 35 |  |
| 28 | Łódź, Masovian Plain, Poland | 1 *E. serotinus* | -80.5 | 210 |  |
| 29 | Wiślańska cave, Beskidy Mountains, Poland | 19 *P. auritus* | -94.1 ± 12.0 | 900 |  |
| 30 | Breziny, Slovakia | 1 *E. serotinus* | -71.9 | 520 |  |
|  | Zvolen, Slovakia | 3 *P. auritus* | -88.3 ± 6.6 | 300 |  |
| 31 | Őcsény, Hungary | 1 *B. barbastellus* | -74.1 | 85 |  |
|  | Szekszárd, Hungary | 9 *E. serotinus*  1 *P. austriacus* | -74.2 ± 5.9  -72.8 | 90 |  |

Name of location, site number (refers to Figure 1), number of individuals of each species captured at each site, mean stable-hydrogen isotopic ratios (‰ relative to VSMOW-SLAP), ± standard deviations when N>3, per species and site, and site elevation.
