# Supplementary material for: A Triple-Isotope Approach to Predict the Breeding Origins of European Bats
Source: PLoS One. 2012 Jan 23;7(1):e30388. doi: 10.1371/journal.pone.0030388 (PMC3264582; doi:10.1371/journal.pone.0030388)
Supplement: Table S2 — Results of the likelihood ratio test (L) for finding the optimal random structure of the linear mixed-effects model (LME) for predicting δDh from δDp. All models were estimated by REML. (DOC) [file pone.0030388.s004.doc]

**Table S2.** Results of the likelihood ratio test (L) for finding the optimal random structure of the linear mixed-effects model (LME) for predicting D_h_ from D_p_.

| **Model** | **Model** | **df** | **AIC** | **logLik** | **L-Ratio** | ***P*** |
| --- | --- | --- | --- | --- | --- | --- |
| (a) | Fixed effect model fitted with GLS | 3 | 1333.49 | -663.75 |  |  |
| (a1) | LME with species as random intercept | 4 | 1331.97 | -661.98 | 3.52 | 0.03 |
| (a2) | LME with species as random slope and intercept | 6 | 1335.46 | -661.73 | 0.51 | 0.62 |

All models were estimated by REML.
