# Supplementary material for: A Triple-Isotope Approach to Predict the Breeding Origins of European Bats
Source: PLoS One. 2012 Jan 23;7(1):e30388. doi: 10.1371/journal.pone.0030388 (PMC3264582; doi:10.1371/journal.pone.0030388)
Supplement: Table S3 — Results of the discriminant function analysis to test the utility of a triple isotope approach for discriminating sampling locations of Eptesicus sp. A. Standardized coefficients given to δD, δ13C and δ15N to maximize the difference between locations. B. Structure matrix, pooled within-groups correlations between discriminating variables and standardized canonical discriminant functions. Variables ordered by absolute size of correlation within function. *. Largest absolute correlation between each variable and any discriminant function. C. Functions at group centroids; unstandardized canonical discriminant functions evaluated at group means. D. Results of the classification in leave-one-out cross-validation, showing how many cases from each location were predicted to stem from each other location, and the total percentage of correctly classified samples per location. For all locations, 93.0% of cross-validated grouped cases were correctly classified. (DOC) [file pone.0030388.s005.doc]

**Table S3.** Results of the discriminant function analysis to test the utility of a triple isotope approach for discriminating sampling locations of *Eptesicus* sp.

| **A. Standardized canonical discriminant function coefficients** | | | | | | | |
| --- | --- | --- | --- | --- | --- | --- | --- |
|  | | Function | | | | | |
|  |  | **1** | | **2** | | **3** | |
| δD | | -0.256 | | 1.013 | | -0.382 | |
| δ^13^C | | 0.574 | | 0.010 | | 0.957 | |
| δ^15^N | | 0.923 | | 0.210 | | -0.396 | |
| **B. Structure matrix** | | | | | | | |
|  | | Function | | | | | |
|  |  | **1** | | **2** | | **3** | |
| δ^15^N | | 0.857^*^ | | 0.022 | | -0.514 | |
| δD | | -0.180 | | 0.979^*^ | | 0.098 | |
| δ^13^C | | 0.283 | | 0.400 | | 0.872^*^ | |
| **C. Functions at group centroids** | | | | | | | |
| Location | | Function | | | | | |
|  |  | **1** | | **2** | | **3** | |
| 2 | | 3.189 | | 1.612 | | 0.134 | |
| 3 | | -3.218 | | 0.362 | | -1.348 | |
| 6 | | 1.440 | | -2.533 | | -2.499 | |
| 14 | | -4.084 | | 0.330 | | 1.487 | |
| 31 | | 1.914 | | -3.382 | | 0.943 | |
| **D. Classification results** | | | | | | | |
| Location | Predicted group membership | | | | | |  |
|  | 2 | 3 | 6 | 14 | 31 | | % Correct |
| 2 | 20 | 0 | 0 | 0 | 0 | | 100 |
| 3 | 0 | 12 | 0 | 1 | 0 | | 92.3 |
| 6 | 0 | 0 | 4 | 0 | 0 | | 100 |
| 14 | 0 | 1 | 0 | 10 | 0 | | 90.9 |
| 31 | 0 | 0 | 2 | 0 | 7 | | 77.8 |

A. Standardized coefficients given to δD, δ^13^C and δ^15^N to maximize the difference between locations. B. Structure matrix, pooled within-groups correlations between discriminating variables and standardized canonical discriminant functions. Variables ordered by absolute size of correlation within function. *. Largest absolute correlation between each variable and any discriminant function. C. Functions at group centroids; unstandardized canonical discriminant functions evaluated at group means. D. Results of the classification in leave-one-out cross-validation, showing how many cases from each location were predicted to stem from each other location, and the total percentage of correctly classified samples per location. For all locations, 93.0% of cross-validated grouped cases were correctly classified.
